# Universal genetic testing for women with newly diagnosed breast cancer in the context of multidisciplinary team care

**DOI:** 10.5694/mja2.51906

**Published:** 2023-04-02

**Authors:** Dilanka L De Silva, Lesley Stafford, Anita R Skandarajah, Michelle Sinclair, Lisa Devereux, Kirsten Hogg, Maira Kentwell, Allan Park, Luxi Lal, Magnus Zethoven, Madawa W Jayawardana, Fiona Chan, Phyllis N Butow, Paul A James, G Bruce Mann, Ian G Campbell, Geoffrey J Lindeman

**Affiliations:** ^1^ The University of Melbourne Melbourne VIC; ^2^ Parkville Familial Cancer Centre Peter MacCallum Cancer Centre and Royal Melbourne Hospital Melbourne VIC; ^3^ Memorial Sloan Kettering Cancer Center New York NY United States of America; ^4^ The Royal Melbourne Hospital Melbourne VIC; ^5^ Royal Women's Hospital Melbourne VIC; ^6^ Peter MacCallum Cancer Centre Melbourne VIC; ^7^ Walter and Eliza Hall Institute of Medical Research Melbourne VIC; ^8^ The Royal Children's Hospital Melbourne Melbourne VIC; ^9^ Centre for Medical Psychology and Evidence‐based Decision Making, the University of Sydney Sydney NSW

**Keywords:** Breast neoplasms, Cancer genes, Genetic testing, Sequence analysis, Genetic counselling, Cancer, Pathology, molecular

## Abstract

**Objective:**

To determine the feasibility of universal genetic testing of women with newly diagnosed breast cancer, to estimate the incidence of pathogenic gene variants and their impact on patient management, and to evaluate patient and clinician acceptance of universal testing.

**Design, setting, participants:**

Prospective study of women with invasive or high grade *in situ* breast cancer and unknown germline status discussed at the Parkville Breast Service (Melbourne) multidisciplinary team meeting. Women were recruited to the pilot (12 June 2020 – 22 March 2021) and expansion phases (17 October 2021 – 8 November 2022) of the Mutational Assessment of newly diagnosed breast cancer using Germline and tumour genomICs (MAGIC) study.

**Main outcome measures:**

Germline testing by DNA sequencing, filtered for nineteen hereditary breast and ovarian cancer genes that could be classified as actionable; only pathogenic variants were reported. Surveys before and after genetic testing assessed pilot phase participants’ perceptions of genetic testing, and psychological distress and cancer‐specific worry. A separate survey assessed clinicians’ views on universal testing.

**Results:**

Pathogenic germline variants were identified in 31 of 474 expanded study phase participants (6.5%), including 28 of 429 women with invasive breast cancer (6.5%). Eighteen of the 31 did not meet current genetic testing eligibility guidelines (probability of a germline pathogenic variant ≥ 10%, based on CanRisk, or Manchester score ≥ 15). Clinical management was changed for 24 of 31 women after identification of a pathogenic variant. Including 68 further women who underwent genetic testing outside the study, 44 of 542 women carried pathogenic variants (8.1%). Acceptance of universal testing was high among both patients (90 of 103, 87%) and clinicians; no decision regret or adverse impact on psychological distress or cancer‐specific worry were reported.

**Conclusion:**

Universal genetic testing following the diagnosis of breast cancer detects clinically significant germline pathogenic variants that might otherwise be missed because of testing guidelines. Routine testing and reporting of pathogenic variants is feasible and acceptable for both patients and clinicians.

**The known:** Medicare‐funded genetic testing is available to people with newly diagnosed breast cancer if the likelihood of finding a pathogenic germline variant, based on family history and tumour pathology, is at least 10%.

**The new:** Germline testing identified pathogenic variants in 31 of 474 women with newly diagnosed invasive breast cancer (6.5%), eighteen of whom did not meet the 10% risk criterion for guideline‐based testing.

**The implications:** Routine testing removes barriers to testing, identifies additional women with pathogenic variants, leading to revised treatment for many, and enables care for family members at risk, all without psychological harm to the tested women.

For people with newly diagnosed breast cancer, identification of a germline (heritable) mutation in a cancer predisposition gene, such as *BRCA1* or *BRCA2*, has important treatment implications. It can inform decisions about therapy, guide future cancer prevention strategies, and facilitate risk management of their blood relatives.^1^

According to Australian guidelines, women should be tested for germline mutations (now termed “pathogenic variants”)^2^ only when their risk of having a pathogenic variant is 10% or greater,^3^ as ascertained with algorithms such as CanRisk^4^ or the Manchester score, which take age, tumour pathology, and family history into account.^5^ In Australia, the Medicare Benefits Schedule (MBS) funds genetic testing for people at this risk threshold.^6^ As demand for testing has grown, some familial cancer centres have introduced “mainstreaming” of people at high risk, in which treating clinicians initiate genetic testing and deliver the result, and familial cancer centres become involved, when required, only after the result is known.^7^, ^8^ In other models, the test result is always delivered by the genetics team.^9^, ^10^ However, it has been reported that many people carrying pathogenic variants might be missed were selection for testing to rely solely on the threshold in the current guidelines.^11^, ^12^

Significantly reduced DNA sequencing costs and increased testing capacity raises the possibility of universal germline testing of women with newly diagnosed breast cancer.^11^ However, more rapid delivery of results would be required to incorporate them into management. Identified variants of uncertain significance may also be challenges; although not requiring clinical action, they can increase anxiety and lead to inappropriate treatment.^6^ While mainstream testing of people with high‐risk breast cancer appears acceptable to both patients and clinicians,^13^, ^14^ the acceptability and psychosocial impact of universal genetic testing are unknown.

The MAGIC (Mutational Assessment of newly diagnosed breast cancer using Germline and tumour genomICs) study examined whether universal germline (and tumour) testing is feasible, clinically useful, and acceptable to women and clinicians in Australia. The primary aim was to determine the proportion of women with either invasive breast cancer or high‐grade non‐invasive disease who carry pathogenic variants, and the proportion missed by selective testing. Secondary objectives included assessing the feasibility of delivering results within eight weeks of testing and assessing their impact on clinical management. The acceptability and psychological impact of universal testing for patients and acceptance by clinicians were assessed in surveys.

## Methods

The MAGIC study commenced as a pilot/feasibility study (157 participants), including a psychosocial sub‐study (12 June 2020 – 22 March 2021); it was followed by an expansion phase with 317 additional participants to improve confidence in estimates of the frequency of pathogenic genetic variants (17 October 2021 – 8 November 2022). All women with newly diagnosed invasive breast cancer, high grade ductal carcinoma *in situ*, or pleomorphic lobular carcinoma *in situ* whose cases were discussed at the weekly multidisciplinary team meeting of the Parkville Breast Service in Melbourne (Peter MacCallum Cancer Centre, Royal Melbourne Hospital, Royal Women's Hospital) were eligible for participation, irrespective of age and tumour phenotype. New diagnosis was defined as self‐ or screen‐detected primary breast cancer, a metachronous cancer, or local recurrence. Exclusion criteria were metastatic cancer, having a previously identified hereditary breast or ovarian cancer pathogenic variant, and previous germline (panel) testing. Eligible women were invited by their treating clinician to participate at one of the seven outpatient clinics in the three hospitals, and blood (or saliva) for germline testing was collected from those who consented. Formalin‐fixed, paraffin‐embedded tumour tissue from a core biopsy or resected tumour was obtained for somatic genetic testing.

In the pilot phase, three‐generation pedigrees were constructed and the CanRisk and Manchester scores calculated for each woman; in the expansion phase, the same was undertaken for women with pathogenic gene variants. Participants for whom the probability of a germline pathogenic variant was at least 10% (calculated using CanRisk, or a Manchester score of at least 15) were considered eligible for Medicare‐funded testing. If a pathogenic variant was detected, eligibility for genetic testing according to the recently updated United States National Comprehensive Cancer Network (NCCN) guidelines (5% risk threshold)^15^ was also determined.

Whole genome sequencing of germline DNA (30× depth) for the pilot phase and whole exome sequencing (100× depth) for the expansion phase were performed. Sequencing (150 base pair paired‐end reads) was performed using the Illumina NovaSeq 6000 and the data were analysed at the Peter Mac Bioinformatics core facility (Peter MacCallum Cancer Centre) to identify all germline and somatic pathogenic variants according to American College of Medical Genetics/Association for Molecular Pathology criteria,^2^ as well as tumour copy number alterations. Germline data were assessed for pathogenic variants in nineteen hereditary breast and ovarian cancer genes (*BRCA1, BRCA2, PALB2, ATM, CHEK2, BARD1, BRIP1, RAD51B, RAD51C, RAD51D, MLH1, MSH2, MSH6, PMS2, CDH1, PTEN, STK11, TP53*, *NTHL1*) that could be classified as actionable (frameshift, nonsense, essential splice site mutations; any missense variants unequivocally deemed pathogenic [class 4 or 5] in the National Library of Medicine ClinVar database, https://www.ncbi.nlm.nih.gov/clinvar). Class 3 variants of uncertain significance were not reported, consistent with the growing preference to not report most variants of uncertain significance,^16^ but highly suspicious (“hot”, “warm”) variants were catalogued separately. The results of tumour genomic sequencing (and detailed psychometric findings) will be reported in a separate publication.

Test results were provided to the treating clinicians and conveyed to the patient by the clinician or a member of the study team. Women with pathogenic variants were referred to the Parkville Familial Cancer Centre for genetic counselling and confirmatory testing. Cases in which a pathogenic variant was identified were re‐presented at the Parkville Breast Service multidisciplinary team meeting, the clinical implications assessed, and any recommended change in management documented.

A clinic audit was also conducted to identify women whose cases were presented at the multidisciplinary team meeting who had elected to separately pursue genetic testing during the study period, either through mainstream testing or Familial Cancer Centre referral (for clinic or self‐funded testing).

### Psychosocial surveys

Each participant in the pilot phase received a baseline (T0) online or paper survey that assessed their knowledge, expectations, and perceptions of genetic testing, and also included validated measures of psychological distress (Depression Anxiety Stress Scale Short Form 21; DASS‐21^17^) and cancer‐specific worry (adapted without validation from the Concerns about Recurrence Questionnaire; CARQ^18^).

A follow‐up (T1) survey (online or paper) including the DASS‐21 and CARQ items was completed two to four weeks after a negative test result, or after a Familial Cancer Centre appointment following a positive result. Overall acceptability was assessed with a single question about whether genetic testing should be offered routinely to all women with breast cancer. Decisional regret was assessed with one question from the Decision Regret Scale^19^ (Supporting Information, part 1).

At the conclusion of the pilot phase, an anonymous online survey that assessed their views on universal germline testing was forwarded by email to all clinicians participating in MAGIC, apart from the study investigators (Supporting Information, part 2).

### Statistical analysis

Data were collected and managed with REDCap electronic data capture tools^20^ and analysed in SPSS 28 (IBM). The statistical significance of changes in DASS‐21 and CARQ scores between the T0 and T1 surveys was assessed in Wilcoxon signed rank tests; a mean change in CARQ score of one‐half of a standard deviation was interpreted as statistically significant.^18^ *P* < 0.05 was deemed statistically significant.

### Ethics approval

The Peter MacCallum Cancer Centre Human Research Ethics Committee provided multi‐site institutional ethics approval (19/224, HREC/58844/PMCC‐2019). Governance approval was obtained from each participating hospital.

## Results

Of 175 women invited to participate in the pilot study, 157 agreed to do so (89.7%) (Box 1). In the expanded phase of the study, the median age of the 474 participants was 55 years (range, 26–91 years; interquartile range [IQR], 47–66 years); 185 women reported family histories of breast cancer (39.0%). The tumour features of the participant group were similar to those typically reported in studies of women with breast cancer.^21^ Sixty‐five women (14%) had received neoadjuvant chemotherapy followed by surgery. Surgical management (prior to germline genetic testing results) was breast conservation for 297 women (62.6%), mastectomy for 170 (35.9%), and nodal excision or chest wall excision for local breast cancer recurrence for seven women (2%) (Box 2).

Box 1 **Recruitment of women to the pilot study, psychosocial sub‐study, and expansion phase of the Mutational Assessment of newly diagnosed breast cancer using Germline and tumour genomICs (MAGIC) study**

**Figure:**
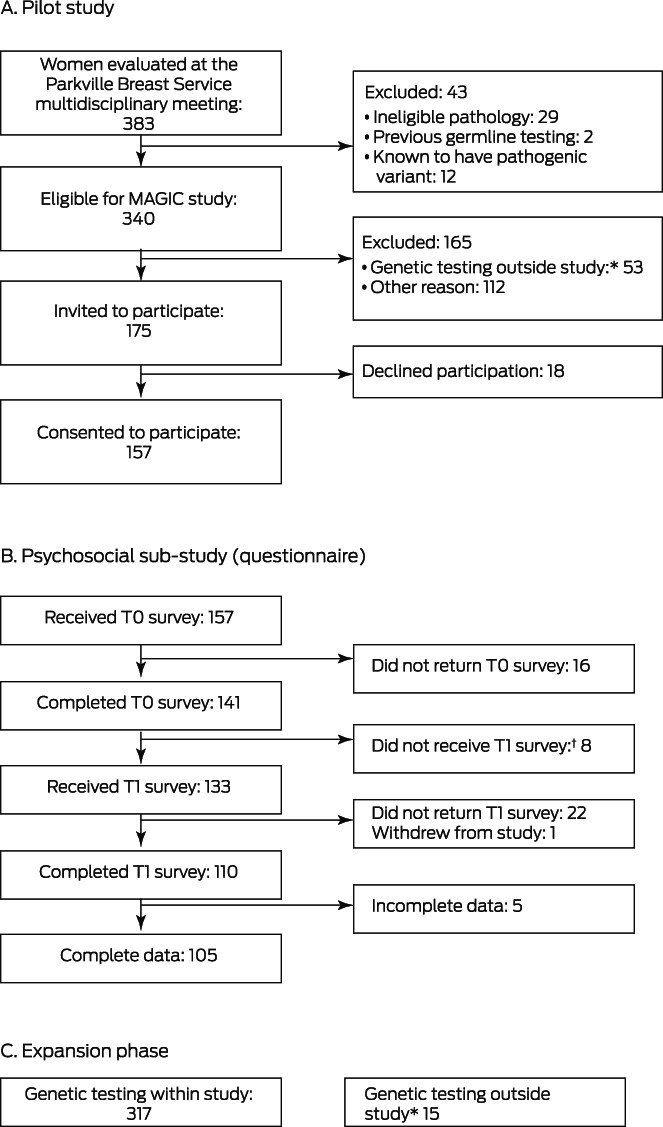
T0 = baseline questionnaire; T1 = post‐genetic test result questionnaire. * Underwent genetic testing outside the study (mainstream testing, Medicare‐funded testing, or self‐funded testing in the Parkville Familial Cancer Centre). † T1 not provided because genetic result had not yet been received.

Box 2 **Characteristics of the 474 women who participated in the Mutational Assessment of newly diagnosed breast cancer using Germline and tumour genomICs (MAGIC) study**

**Table:** 

Characteristic	All participants	Pathogenic variant identified*	No pathogenic variant identified
Number of women	474	31 [6.5%]	443 [93.5%]
Age (years), median (IQR)	55.5 (47–66)	57.0 (50–64)	55.0 (47–66)
Age (years), range	26–91	36–83	26–91
Cancer phenotype			
Invasive ductal	344 (72.5%)	26 (83.9%)	318 (71.8%)
Invasive lobular	61 (13%)	1 (3%)	60 (14%)
Special types	24 (5.1%)	1 (3%)	23 (5.2%)
High grade ductal carcinoma *in situ*/pleomorphic lobular carcinoma *in situ*	45 (9.5%)	3 (10%)	42 (9.5%)
Breast surgery			
Breast conservation	297 (62.6%)	15 (48.4%)	282 (63.6%)
Mastectomy	170 (35.9%)	16 (51.6%)	154 (34.8%)
Other (chest wall or node excision)	7 (2%)	0	7 (2%)
Family history of breast cancer			
Yes	185 (39.0%)	18 (58.0%)	167 (37.7%)
No	261 (55.1%)	11 (35.5%)	250 (56.4%)
Unknown	28 (5.9%)	2 (6%)	26 (5.9%)
Invasive cancer: phenotype	429	28	401
ER+ HER2–	317 (73.9%)	19 (68%)	298 (74.3%)
HER2+ (ER+)	51 (12%)	4 (14%)	47 (11.7%)
HER2+ (ER–)	19 (4.4%)	1 (4%)	18 (4.5%)
Triple negative breast cancer	42 (9.8%)	4 (14%)	38 (9.5%)
Invasive cancer: stage			
1	170 (39.6%)	12 (43%)	158 (39.4%)
2	124 (28.9%)	8 (29%)	116 (28.9%)
3	48 (11%)	1 (4%)	47 (12%)
Not assessable^†^	87 (20%)	7 (25%)	80 (20%)

ER+ = oestrogen receptor‐positive; ER– = oestrogen receptor‐negative; HER2+ = HER2‐positive (amplified); HER2– = HER2‐negative (non‐amplified).

* Differences between participants with or without pathogenic variants were not statistically significant, except for family history (Fisher exact test: *P* = 0.030).

^†^ Sixty‐five women receiving neoadjuvant chemotherapy, 22 without primary/nodal assessment.

A germline pathogenic variant (in *BRCA1, BRCA2, PALB2, CHEK2, ATM, RAD51C, BARD1, PMS2*, or *MSH6*) was identified in 31 of 474 women (6.5%), including 28 of 429 women with invasive breast cancer (6.5%) (Supporting Information, table). The median time from testing to result notification was 44 days (IQR, 37–56 days). Eighteen women with pathogenic variants (58%) reported family histories of breast cancer, as did 167 of those without pathogenic variants (38%); the median ages of the two groups were similar (Box 2).

Two algorithms were used to determine the likelihood of finding a pathogenic variant (and therefore eligibility for Medicare‐funded testing). CanRisk would have identified ten of the 31 women with pathogenic variants (32%) and Manchester scores eight (26%); if both algorithms were used, thirteen women with pathogenic variants (42%) would have been eligible for Medicare‐funded testing (Box 3; Supporting Information, table). Six of the 31 women with pathogenic variants (19%) did not reach the 5% risk threshold of the recently updated NCCN guidelines^15^ (Supporting Information, table).

Box 3 Classification of 31 participants found to carry pathogenic gene variants, by gene, estimated risk of having a pathogenic gene variant,* and effect on treatment recommendations

**Figure:**
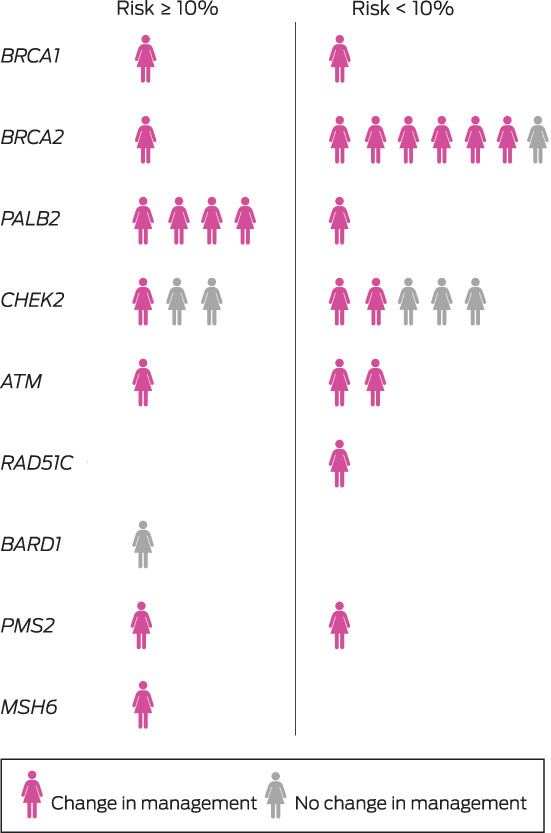
* Estimated with the CanRisk or Manchester algorithms. Further information is included in the Supporting Information table.

The multidisciplinary team modified their treatment recommendations for 24 of the 31 women with pathogenic variants (77%), including fourteen of the eighteen not eligible for Medicare‐funded testing (Box 3; Supporting Information, table). The modified recommendations included consideration of contralateral or bilateral mastectomy for fifteen women. Eighteen were referred for consideration of risk‐reducing bilateral salpingo‐oophorectomy (and hysterectomy for women with *PMS2* and *MSH6* variants), and three women with pathogenic *ATM* variants were closely monitored for radiation toxicity.

Our clinic audit identified a further 68 women discussed at the multidisciplinary team meeting who pursued genetic testing outside the study, 56 of whom (82%) were eligible for publicly funded testing (consistent with their known increased risk). Of these, thirteen women were found to carry pathogenic variants (19%). In total, 44 of the 542 women who opted for genetic testing at the time of their breast cancer diagnoses (8.1%), including 41 of 497 with invasive breast cancer (8.2%), had clinically significant pathogenic variants.

### Psychosocial outcomes for tested women

A total of 105 women provided complete T0 and T1 follow‐up survey data (median age, 56 years; range, 26–78 years; IQR, 48–66 years), including 96 of 145 pilot phase participants negative (66%) and nine of twelve positive for pathogenic variants (75%). Thirty‐eight respondents had university qualifications, 23 spoke languages other than English at home, 80 had children, 27 had received formal mental health interventions, and a further fourteen were receiving mental health interventions at T0.

Ninety of 103 respondents agreed or strongly agreed that all women with breast cancer should be offered genetic testing; thirteen were neutral, none disagreed. One hundred of 104 respondents believed their decision to undergo genetic testing had been correct, four were neutral, and none disagreed. Median cancer‐specific distress declined from 13 (IQR, 6.0–29.5) points at T0 to 10 (IQR, 4.0–21.5) points at T1 (*P* < 0.001). Levels of cancer‐specific distress remained unchanged for 47 respondents, declined for 41 respondents (median change, 11.0 [IQR, 7.5–17.5] points), and increased for seventeen respondents, fifteen of whom had negative and two positive germline test results (median change, 13.0 [IQR, 7.0–17.0] points). Median cancer‐specific distress was similar at T1 for women with negative (10.0 points; IQR, 4.3–21.8 points) or positive test results (8.0 [IQR, 0.0–20.0] points; *P* = 0.027), as were the proportions of respondents with greater distress at T1 (data not shown). Changes in symptoms of depression, anxiety, and stress between T0 and T1 were not statistically significant (data not shown).

### Acceptability of routine testing for clinicians and impact on clinical practice

Twenty‐five (fourteen women, eleven men) of thirty‐three clinicians completed the clinician survey: eleven of fourteen breast surgeons, eight of nine medical oncologists, four of six breast care nurses, and two of four radiation oncologists. All reported that genetic test results were helpful for important treatment decisions, and none that testing was distressing for their patients. After excluding responses from breast care nurses (who cannot refer patients for genetic testing), eight of the 21 respondents said that, prior to participation in MAGIC, they frequently referred women for germline testing for whom it was not publicly subsidised; after participating in MAGIC, fourteen of these respondents were more likely and seven as likely as before participation to refer women for germline testing when criteria for publicly subsidised testing were not met. Were all germline testing subsidised, seventeen of 21 respondents agreed or strongly agreed that they would offer it to each woman during their first consultation rather than selecting candidates according to histology and family history.

## Discussion

Identifying pathogenic germline variants is clinically important for optimising surgical and other management of breast cancer, including targeted therapy.^22^ Genetic testing guidelines, however, can lead to under‐detection of pathogenic variants.^11^, ^12^, ^23^ In our prospective study, we found that pathogenic germline variants were identified in 6.5% of women with invasive breast cancer, and that eighteen of the 31 women with clinically significant and actionable germline pathogenic variants would not have been tested under the current guidelines.

The underlying rate of pathogenic germline variants in the population from which our sample was drawn is probably greater than 6.5%. Firstly, we excluded twelve women already known to have pathogenic variants. Secondly, a clinic audit of women who elected to pursue genetic testing outside the study found that thirteen of 68 had pathogenic variants. Consequently, 8.1% of all women who opted for genetic testing after diagnosis had clinically significant pathogenic variants.

Test results were returned to clinicians after a median of 44 days. More rapid turnaround, which could assist surgical decisions, would require more resources. Nevertheless, the current turnaround time would enable implementation of recent findings from the OlympiA study, in which a subset of patients with *BRCA1* or *BRCA2* pathogenic variants benefited from adjuvant therapy with the poly (ADP‐ribose) polymerase (PARP) inhibitor olaparib.^22^

Universal testing was strongly endorsed by our demographically diverse group of women with breast cancer and was not associated with decisional regret, increased cancer‐specific worry, or changes in depression, anxiety, or stress. Clinicians unanimously reported that their patients were not distressed, and that they supported universal testing. About 90% of women invited to participate in the MAGIC study did so, suggesting that universal germline testing for people with newly diagnosed invasive breast cancer would be broadly accepted.

Offering clinician‐led testing to all women with newly diagnosed breast cancer probably removed barriers to testing, particularly for culturally and linguistically diverse people less able to negotiate multiple appointments. Moreover, preparing a detailed three‐generation pedigree to determine funding eligibility can be inaccurate if undertaken outside a familial cancer centre, and referring all women with newly diagnosed disease to our familial cancer centre would have been expensive and overwhelmed the service. Improving the ability of clinicians to explain genetic information to patients was readily achieved, leaving the Familial Cancer Centre to focus on women with pathogenic variants and to initiate cascade testing of asymptomatic family members to determine their carrier status.

MAGIC did not report variants of uncertain significance (class 3), avoiding needless anxiety for women with non‐actionable variants, placing undue burden on familial cancer centres, and unnecessary treatment. Despite discussions of the ethical and psychosocial implications of reporting (or not reporting) these variants, only about 10% of uncertain variants that are reclassified prove to be pathogenic (class 4 or 5).^24^ Variants of uncertain significance are identified in 8.7% of women who undergo mainstream testing at our centre;^7^ even were all these variants reclassified, the proportion of tested women reported to have pathogenic variants would increase by only one percentage point. This is a much smaller proportion than the 58% of certainly pathogenic variants missed by applying the current testing guidelines. Reporting hot or warm variants of uncertain significance (which may be reclassified as pathogenic), however, is important and consistent with more recent guidelines.^16^ No such variants were identified in our sample, suggesting that they are infrequent.

### Limitations

Recruitment was hampered by the COVID‐19 pandemic, during which both women and their clinicians faced unique challenges beyond the usual complexity of a new diagnosis. Some eligible women were consequently not invited to participate in MAGIC, raising the possibility that our findings are not broadly generalisable.

We assessed nineteen hereditary breast and ovarian cancer genes, including Lynch syndrome genes, which are not associated with increased breast cancer risk.^25^ We identified one *MSH6* and two *PMS2* pathogenic variants that were clinically actionable but did not influence breast cancer management; were these two genes removed and ten key breast cancer susceptibility genes considered,^26^ pathogenic variants would still have been identified in 41 of 542 tested women (7.6%).

Our study did not evaluate the economic costs and benefits of universal genetic testing. Financial modelling in the United Kingdom and the United States indicated that unselected multi‐gene testing for all women with breast cancer is extremely cost‐effective compared with testing based on family history or clinical criteria.^27^ About 20 640 people were diagnosed with breast cancer in Australia in 2022.^28^ Based on the current Medicare rebate, universal genetic testing would cost about $25 million per year, but the opportunity costs of missing pathogenic variants in more than 50% of cases by applying current guidelines is unknown.

### Conclusion

The MAGIC study found that universal germline testing of women with newly diagnosed breast cancer as part of multidisciplinary care was both feasible and acceptable, regardless of patient age, family history, and tumour pathology. It led to the detection of many instances of actionable germline pathogenic variants that would have been missed using current testing guidelines. Universal germline testing could optimise the management of women with breast cancer and the management of risk for their family members.

## Open access

Open access publishing facilitated by The University of Melbourne, as part of the Wiley ‐ The University of Melbourne agreement via the Council of Australian University Librarians.

## Competing interests

No relevant disclosures.

## Supporting information


Supplementary methods and results
