# Supplementary material for: Universal genetic testing for women with newly diagnosed breast cancer in the context of multidisciplinary team care
Source: Med J Aust. 2023 Apr 2;218(8):368–73. doi: 10.5694/mja2.51906 (PMC10952347; doi:10.5694/mja2.51906)
Supplement: Supplementary file 1 — Supplementary methods and results [file MJA2-218-368-s001.pdf]

The Medical Journal of Australia

Supporting Information

Supplementary methods and results

This appendix was part of the submitted manuscript and has been peer reviewed.
It is posted as supplied by the authors.

Appendix to: De Silva DL, Stafford L, Skandarajah, AR, et al. Universal genetic testing for women with newly diagnosed breast cancer in the context of multidisciplinary team care. Med J Aust 2023; doi: 10.5694/mja2.51906.

1. Investigator-developed study-specific questions and measures that have been adapted for use in this study for completion by patient participants.
Cancer-specific worry measure adapted from the Concerns about Recurrence Questionnaire (CARQ)

For each question, please tick the box for the answer that best reflects how you felt in THE PAST WEEK.

1. **How often have you worried about results of your genetic testing?**

0	1	2	3	4	5	6	7	8	9	10

None of the time   All of the time

2. **To what extent does worry about your genetic testing results spill over or intrude on your thoughts and activities?**

0	1	2	3	4	5	6	7	8	9	10

Not at all   A great deal

3. **How emotionally upset or distressed have you been thinking about the results of your genetic testing?**

0	1	2	3	4	5	6	7	8	9	10

Not at all   A great deal

4. **How often have you worried about the possibility developing another cancer/ having a recurrence of breast cancer?**

0	1	2	3	4	5	6	7	8	9	10

None of the time   All of the time

5. **To what extent does worry about having a recurrence of breast or developing another cancer spill over or intruded on your thoughts and activities?**

0	1	2	3	4	5	6	7	8	9	10

Not at all   A great deal

6. **How emotionally upset or distressed have you been thinking about the possibility of having a recurrence of breast cancer or developing another cancer?**

0	1	2	3	4	5	6	7	8	9	10

Not at all A great deal

Overall acceptability of routine genetic testing

Based on your experience, to what extent do you agree that the germline and somatic sequencing (the genetic testing that you had) should be offered to all breast cancer patients in the Australian public health system?

Strongly disagree	Disagree	Neither agree or disagree	Agree	Strongly agree
❑1	❑2	❑3	❑4	❑5

Decisional Regret Scale

Please show how strongly you agree or disagree with this statement by selecting a number from 1 (strongly disagree) to 5 (strongly agree) that best fits your view about your decision to have genetic testing.

	Strongly disagree	Disagree	Neither agree nor disagree	Agree	Strongly agree
a. It was the right decision	❑1	❑2	❑3	❑4	❑5

2. Investigator-developed questions contained in the clinician participant survey.

These questions ask about your participation in the MAGIC study. Please indicate your level of agreement with each statement from *strongly disagree* to *strongly agree*. Please tick the Not Applicable (NA) response if appropriate.

	Strongly Disagree	Disagree	Neither agree nor disagree	Agree	Strongly Agree	Not applicable
1. Genetic test results were helpful in making important treatment decisions						
2. The genetic testing process was distressing for my patients						

These questions ask about the impact of MAGIC on your ongoing clinical practice.

	Never	Sometimes	Occasionally	Frequently	Not applicable
1. Before your experience with the MAGIC study, how often would you refer women with breast cancer for germline testing in the absence of a known family history/when testing was not subsidized? (i.e., patient would need to pay privately for testing testing)					

	Less likely than before	About the same as before	More likely than before	Not applicable
2. Assuming germline testing does not become part of universal care, after your experience with the MAGIC study, **how likely are you now** to offer germline testing in the absence of a known family history/when testing is not subsidized?				

	Strongly disagree	Disagree	Neither agree or disagree	Agree	Strongly agree	Not applicable
3. If publicly funded, I would be happy to offer germline testing at the first consultation for every patient rather than selecting based on histology and family history.						

Table. Germline pathogenic variants (mutations) identified and their impact on management

Participant ID[1]	Pathogenic variant	Age (years)	Tumour grade and phenotype	CanRisk Score[2]	Manchester Score[3]	NCCN[4]	Eligible for MBS-funded testing	Treatment change due to the germline pathogenic variant
2020-018	*PALB2*	67	G3 IDC,<br>ER+ PR– HER2–	3%	2	Yes	No	Referral for RR BSO
2020-023	*CHEK2*	43	Bilateral BC<br>1. G2 IDC,<br>ER+ PR+ HER2–<br>2. HG DCIS	20.7%	16	Yes	Yes	No change in management
2020-026	*BRCA1*	48	G3 IDC,<br>ER+ PR– HER2–	4.4%	8	Yes	No	Bilateral mastectomy and RR BSO
2020-043	*PALB2*	52	Bilateral G2 IDC<br>1. ER+ PR– HER2–<br>2. ER+ PR+ HER2+	21%	16	Yes	Yes	Bilateral mastectomy and RR BSO
2020-050	*PALB2*	61	G3 IDC,<br>TNBC	19.1%	11	Yes	Yes	Bilateral mastectomy and RR BSO
2020-055	*PMS2*	61	G2 Invasive micropapillary,<br>ER+ PR+ HER2–	4.6%	15	Yes	Yes	Gastroenterology and gynaecology referral (for endoscopies, hysterectomy/oophorectomy)
2020-061	*BRCA2*	51	G3 IDC,<br>ER+ PR+ HER2–	6.1%	5	No	No	Bilateral mastectomy and RR BSO
2020-120	*CHEK2*	72	G1 IDC,<br>ER+ PR+ HER2–	3.1%	-1	No	No	No change in management
2020-127	*ATM*	36	G2 IDC,<br>ER+ PR+ HER2–	9.9%	9	Yes	No	Close monitoring for radiation toxicity[5]
2020-137	*PMS2*	48	G3 IDC,<br>ER– PR– HER2+	5.3%	5	Yes	No	Gastroenterology and gynaecology referral (for endoscopies, hysterectomy/oophorectomy)[6]
2020-153	*BRCA1*	58	G3 IDC<br>TNBC	12.9%	16	Yes	Yes	Bilateral mastectomy and RR BSO

Participant ID[1]	Pathogenic variant	Age (years)	Tumour grade and phenotype	CanRisk Score[2]	Manchester Score[3]	NCCN[4]	Eligible for MBS-funded testing	Treatment change due to the germline pathogenic variant
2020-188	*ATM*	61	G3 IDC,<br>ER+ PR+ HER2–	8.7%	14	Yes	No	Close monitoring for radiation toxicity[5]
2021_029	*BRCA2*	40	G3 IDC,<br>ER+ PR+ HER2+	8.2%	7	Yes	No	Bilateral mastectomy and RR BSO
2021_060	*CHEK2*	65	HG DCIS<br>(ER+ PR–)	3.9%	10	Yes	No	No change in management
2021_077	*BRCA2*	54	G3 IDC,<br>ER+ PR+ HER2–	6.5%	6	Yes	No	Bilateral mastectomy and RR BSO
2021_104	*CHEK2*	46	G2 IDC,<br>ER+ PR+ HER2–	7.8%	15	Yes	Yes	Bilateral mastectomy[7]
2021_143	*BRCA2*	53	G3 IDC,<br>ER– PR– HER2+	2.5%	2	No	No	Bilateral mastectomy and RR BSO; Avoided radiation therapy
2021_153	*BRCA2*	83	G3 IDC,<br>ER+ PR– HER2–	3.4%	3	Yes	No	No change in management
2021_188	*PALB2*	50	G3 IDC,<br>TNBC	10.4%	11	Yes	Yes	Bilateral mastectomy and RR BSO; Avoided radiation therapy
2021_189	*RAD51C*	69	HG DCIS<br>(ER+ PR–)	4%	-7	No	No	Referral for RR BSO
2021_241	*BARD1*	78	G2 ILC,<br>ER+ PR+ HER2–	14.8%	27	Yes	Yes	No change in management
2021_295	*PALB2*	63	G2 IDC,<br>ER+ PR+ HER2–	19.2%	11	Yes	Yes	Bilateral mastectomy and RR BSO
2021_303	*CHEK2*	63	G3 IDC,<br>ER+ PR+ HER2–	16.6%	10	Yes	Yes	No change in management
2021_310	*MSH6*	60	G3 IDC,<br>ER+ PR+ HER2–	12.6%	17	Yes	Yes	Gastroenterology and gynaecology referral (for endoscopies, oophorectomy; prior hysterectomy)

Participant ID[1]	Pathogenic variant	Age (years)	Tumour grade and phenotype	CanRisk Score[2]	Manchester Score[3]	NCCN[4]	Eligible for MBS-funded testing	Treatment change due to the germline pathogenic variant
2021_327	*CHEK2*	57	HG DCIS (ER– PR–)	4.8%	9	Yes	No	Bilateral mastectomy[7]
2021_345	*BRCA2*	54	G3 IDC, ER+ PR+ HER2–	5.1%	5	No	No	Right mastectomy and RR BSO recommended
2021_376	*BRCA2*	74	G2 IDC, TNBC	40%	14	Yes	Yes	RR BSO recommended
2021_379	*ATM*	75	G3 IDC, ER+ PR– HER2+	7.1%	25	Yes	Yes	Mastectomy or close monitoring for radiation toxicity recommended[5]
2021_389	*CHEK2*	43	G3 IDC, ER+ PR+ HER2–	5%	7	Yes	No	Bilateral mastectomy[7]
2021_403	*BRCA2*	61	G3 IDC, ER+ PR+ HER2–	4.4%	3	No	No	Referral for RR BSO
2021_409	*CHEK2*	50	G2 IDC, ER+ PR+ HER2–	8.7%	4	Yes	No	No change in management

BC, breast cancer; G, grade; IDC, invasive ductal cancer; ILC, invasive lobular cancer; ER, oestrogen receptor; PR, progesterone receptor; HER2+, HER2 amplified; HG DCIS, High-Grade Ductal Carcinoma in Situ; TNBC, triple negative breast cancer, RR BSO, risk reducing bilateral salpingo-oophorectomy; MBS, Medicare Benefits Schedule.

[1] Pilot study ID 2020, expansion phase ID 2021.

[2] CanRisk: probability of identifying a germline mutation. Scores ≥10% are eligible for MBS funded testing

[3] Manchester score: Scores of ≥15 are eligible for MBS funded testing

[4] Would qualify for testing if adopted National Comprehensive Cancer Network (NCCN) guidelines version 2.2023 (>5% probability)

[5] EviQ guidelines indicate mixed reports regarding the effects of radiation on heterozygous *ATM* pathogenic variant carriers but that radiation therapy at conventional doses is not contraindicated and should be considered and delivered if required. The care of affected women should be individualised based on their clinical situation (ID: 1610 v.8)

[6] This woman elected to undergo bilateral mastectomy.

[7] EviQ guidelines indicate that breast cancer risk should be formally assessed using a validated tool like CanRisk and that high-risk management applies when the lifetime risk from age 20 years is 30% or greater, and/or the risk between ages 40-50 years is greater than 8%. (ID: 3701 v.3).

References

CanRisk:

Carver T, Hartley S, Lee A, et al. CanRisk tool-a web interface for the prediction of breast and ovarian cancer risk and the likelihood of carrying genetic pathogenic variants. Cancer Epidemiol Biomarkers Prev 2021; 30: 469-473.

Manchester score:

Evans DG, Lalloo F, Cramer A, et al. Addition of pathology and biomarker information significantly improves the performance of the Manchester scoring system for BRCA1 and BRCA2 testing. J Med Genet 2009; 46: 811-817.

NCCN guidelines

National Comprehensive Cancer Network. Genetic/familial high-risk assessment: breast, ovarian, and pancreatic (NCCN clinical practice guidelines in oncology; version 3.2023). https://www.nccn.org/guidelines/guidelines-detail?category=2&id=1503 (viewed Feb 2023).

ATM

Cancer Institute NSW. ATM (monoallelic pathogenic variants) – risk management (EviQ 1610 v.8). https://www.eviq.org.au/cancer-genetics/adult/risk-management/1610-atm-monoallelic-pathogenic-variants-risk#history (viewed Feb 2023).

CHEK2

Cancer Institute NSW. CHEK2 – risk management (female) (EviQ 3701 v.3). https://www.eviq.org.au/cancer-genetics/adult/risk-management/3701-chek2-risk-management-female#history (viewed Feb 2023).
